# Where Is the Loop-hole

**Published:** 1896-10

**Authors:** 


WHERE IS THE LOOP-HOLE?—It has long been a matter of wonderment, in view of the rules and regulations of the Medical College Association and the alleged "requirements for matriculation and for graduation," published in the medical college catalogues, how certain persons ever obtained a medical diploma. Conferring the degree of doctor of medicine upon certain persons whom we know to be holders of diplomas from reputable medical colleges is farcial, in view of the fact that they are ignorant, have no literary or other education, and really do not know how to spell the simplest words in the English language, nor how to write an order on the corner grocery for a peck of potatoes. To confer the honor of the degree on these fellows is a mockery; to thus license them to deal with life and death is an outrage—almost a crime. We gave two or three sample letters from "doctors" of this sort in our July edition, letters from men who begin a letter with a small letter, and do not capitalize the all important personal pronoun, first person singular, but put it thus, "i," and follow it, perhaps, with a capital "R," and write it "Rite" (and although he may write it "rite," it is, nevertheless, wrong). And the writer of at least one of these letters is personally known to us as a graduate of a prominent medical college, and a member in good standing of the Texas State Medical Association. How do these fellows get through the ordeal? It is very evident that some of the colleges are "playing off" on the Association of colleges, and they ought to be looked after. We suppose that this college, or these colleges, that are graduating such material reason like the Irishman did when the priest requested every member of the congregation to bring a pint of wine and pour it into a cask for a present. One poor follow felt too poor to bring the wine, and he reasoned that as everybody else would pour in a bottle of wine, his little bottle of water would not be detected, and accordingly he added a pint of water. The result demonstrated that everybody in the congregation felt the same way, and hence the priest had a barrel of water for his present. Are all of the colleges disregarding the preliminary examination? Below we give the requirements of both the college associations:

ASSOCIATION OF AMERICAN MEDICAL COLLEGES.

The Association of American Medical Colleges, requires for all members that candidates for matriculation will be allowed admission, subject to the conditions prescribed by Article III of the Constitution of the Association:

ARTICLE III.

SECTION 1.—Members of this Association shall require of all matriculants an English composition in the handwriting of the applicant of not less than two hundred words; an examination by a Committee of the Faculty, or other lawfully constituted Board of Examiners, in higher algebra, elementary physics, and Latin prose.

SEC. 2. Graduates or matriculants of reputable colleges or high schools of the first grade, or normal schools established by State authority, or those who may have successfully passed the entrance examination provided by the statutes of the State of New York, shall be exempt from the requirements of Section 1.

SEC. 3. Students conditioned in one or more of the branches enumerated as requirements for matriculation shall have time until the beginning of the second year to make up such deficiencies; provided, however, that students who fail in any of the required branches in this second examination shall not be admitted to the second course.

SEC. 4. Colleges granting final examination on elementary subjects to junior students shall not issue certificates of such final examination, nor shall any member of this Association confer the degree of Doctor of Medicine upon any person who has not been first examined upon all the branches of the curriculum by the Faculty of the college granting the degree.

SEC. 5. Candidates for the degree of Doctor of Medicine shall have attended three courses of graded instructions of not less than six months each in three separate years.

SEC. 6. Students who have matriculated in any regular college prior to July 1, 1892, shall be exempted from these requirements.

THE SOUTHERN MEDICAL COLLEGE ASSOCIATION.

The Southern Medical College Association makes the following requirements, viz:

Every student applying for matriculation must possess the following qualifications:

He must hold a certificate as the pupil of some known, reputable physician, showing his moral character and general fitness to enter upon the study of medicine.

He must possess a diploma of graduation from some literary or scientific institution of learning, or certificate from some legally constituted high school, general superintendent of State education, or superintendent of some county board of public education, attesting the fact that he is possessed of at least the educational attainments required of second-grade teachers of public schools; provided, however, that if a student so applying is unable to furnish the above and foregoing evidence of literary qualifications, he may be permitted to matriculate and receive medical instruction as other students, and qualify himself in the required literary departments, and stand his required examination, as above specified, prior to offering himself for a second course of lectures.

The foregoing certificate of educational qualifications, attested by the Dean of the medical college attended, together with a set of tickets showing that the holder has attended one full course of medical lectures shall be essential to attendance upon a second course of lectures in any college belonging to the Southern Medical College Association.

---

DR. JOHN B. HAMILTON, the able and popular editor of the *Journal* of the American Medical Association, it seems, is also a surgeon in the Marine Hospital Service, and, as such, is subject to orders of the head of that service. Doubtless General Wyman, the Supervising Surgeon General, has observed the attitude of the Association's *Journal* with reference to the proposed bureau of public health, and made a note on it; put it away for future use, and smoked on it, leisurely. It will be remembered that a short time before his death, Dr. Jerome Cochran, as delegate or representative of his State, at the recent conference of State Boards of Health, got the innings and put through, by stocking the convention, it is said, a resolution to the effect that the profession of medicine of the United States desire a sub-bureau and not a separate and distinct department; and proposed to put said proposed bureau under control of the Marine Hospital Service. This, no doubt, was very much to the liking of Surgeon General Wyman, who, no doubt, instigated Cochran to the trick. When, therefore, the Association's *Journal*, edited by a M. H. S. sub-officer, kicked against it, then it was that Wyman put on his war paint, and went for Hamilton's scalp. He got it, we are sorry to learn; that is, he *ordered* him to *leave Chicago*, leave the *Journal* (big salary, it is said), and take charge of the M. H. S, station at San Francisco!—practically, under the circumstances, Botany Bay! Just how the matter is going to be adjusted to the satisfaction of all, remains to be seen. Seems to me—if we were in J. B.'s place, we would resign the M. H. S.

